# Epidemiology of Gasoline Burn Injuries in Tehran, Iran

**Published:** 2011-03-01

**Authors:** Sh Firoozbakhsh, S Seifirad, V Yamin

**Affiliations:** 1 Pulmonary and Critical Care Research Center,Tehran University of Medical Sciences, Tehran, Iran

**Keywords:** Burn, Epidemiology, Gasoline, Iran

Dear Editor,

Gasoline burn is one of the major causes of thermal burns even in the developed countries. Lack of understanding on explosive nature of gasoline has resulted into its inappropriate and unsafe use. In order to determine the frequency and outcomes of gasoline burns in our center in Tehran, Iran, we reviewed the hospital records of all acute gasoline burns during the last year. Patient’s age groups and their jobs, the mechanism of burn, Total Body Surface Area (TBSA), total hospitalization days, frequency of inhalation injury and mortality during their hospital admission were determined.

One hundred and seventy seven gasoline burned patients were admitted compromising 18% of all acute burn admissions in our center affiliated to Tehran University of Medical Sciences. The Mean TBSA was 46.41%±27.33. The mean hospitalization was 17.77±15.83 days. Eighty six (42.9%) patients had smoke inhalation injuries based upon their history and physical exam. The mean burn percentile was 60.51 ±15.8% in contrast with 33.32±20.18% in those without inhalation injuries (p<0.05).Their mortality rate was 58.1% in contrast with 11% in others. Ninety eight (55.4%) patients were in their third or fourth decade of life and 26.6% were less than 20 years. Distribution of patients by age and TBSA is shown in Table 1. One hundred and twelve (63.2%) patients were workers and 36 (20.3%) were students. Eleven (6.2%) injuries were due to car accidents. Misuse of gasoline as a cleaning solution or an accelerant was responsible for 45% of cases and improper means of transport and storage was the cause of burn in 36%. Suicidal attempt was not well documented; because of loss of insurance coverage in these patients, it was not reported by patients and their kin.

Flame was responsible for 36.4% of male and 43.6% of female burn injuries in Azerbaijan Province, western Iran.[1] Gasoline was also previously reported to be responsible for 24.8% of total burn injuries in south west of Iran.[2] Flame was also the most common etiology of burns (63.7%) in Kurdistan Province, western Iran.[3] Kerosene and Petrol were responsible for approximately 30% of burn injuries in Tabriz, western Iran.[4] High Mortality rate observed in our patients (33.9%) may be related to high prevalence of inhalation injuries. Inhalation injuries are frequently (42.9%) seen in our patients whose total burn surface (60.51%) and mortality rate (58.1%) were higher in comparison with those without this complication (11%). Among 44 dead victims, 36 (81.81%) patients had inhalation injuries. Because of high mortality rate in patients with inhalation injury, the mean hospitalization (13.91±14.96 days) were lower in comparison with the others (21.43±15.83 days).

Surprisingly, no report of burns in relation to carburetor priming was found in our review which was frequently noticed in other studies.[5][6][7] Most of these events could be prevented if the patients were aware of the dangers of gasoline and the proper way of its use. This problem is not limited to our country. Barillo et al. reported that gasoline-related burn injuries were responsible for 23.3% of all acute burn admissions during their 18 years study period. Inhalation injuries were observed in 28.1% of cases and only in 12.9% of injuries, gasoline had been used safe and appropriately.[8] Williams et al. reported that 14.5% of all admissions in their burn center were due to gasoline and the majority (59%) were the result of inappropriate or unsupervised use of gasoline.[9] In a 2-year period study by Wilson and Bailie, nearly 33% of the adult male patients’ admissions were petrol-related.[10] With respect to the frequency and high mortality of gasoline related injuries in our country, we conclude that more information must be available for the public about the hazards of this popular and useful fuel. The high risk groups (i.e. workers) can become aware of these hazards by studying pamphlets about this subject and attending teaching sessions being held at their workplace. Television programs and learning items in students' scheduled programs could play a key role in informing the population of these hazards. The effect of these interventions can be assessed later on.

**Table 1:** Distribution of patients by Age and TBSA.

**TBSA a (%)**	**0-10**	**11-20**	**21-40**	**41-60**	**61-80**	**81-100**	**Total**
Age group (year)							
0-5	0	1	1	1	1	1	5
6-15	0	2	7	3	0	2	14
16-25	0	9	25	11	11	14	70
26-35	3	7	12	7	6	6	41
36-45	3	4	9	6	4	3	29
46-55	1	2	4	1	1	2	11
56-65	0	0	0	0	2	0	2
65 <	1	0	3	0	0	1	5
Total	8	25	61	29	25	29	177

^a^ TBSA: Total Body Surface Area
